# Supplementary material for: Towards unified AI-driven fracture mechanics: the extended deep energy method (XDEM)
Source: Nat Commun. 2026 Aug 15;17:8492. doi: 10.1038/s41467-026-76748-1 (PMC13484471; doi:10.1038/s41467-026-76748-1)
Supplement: Supplementary file 3 [file 41467_2026_76748_MOESM3_ESM.zip › pic/Method/embedding.pdf]

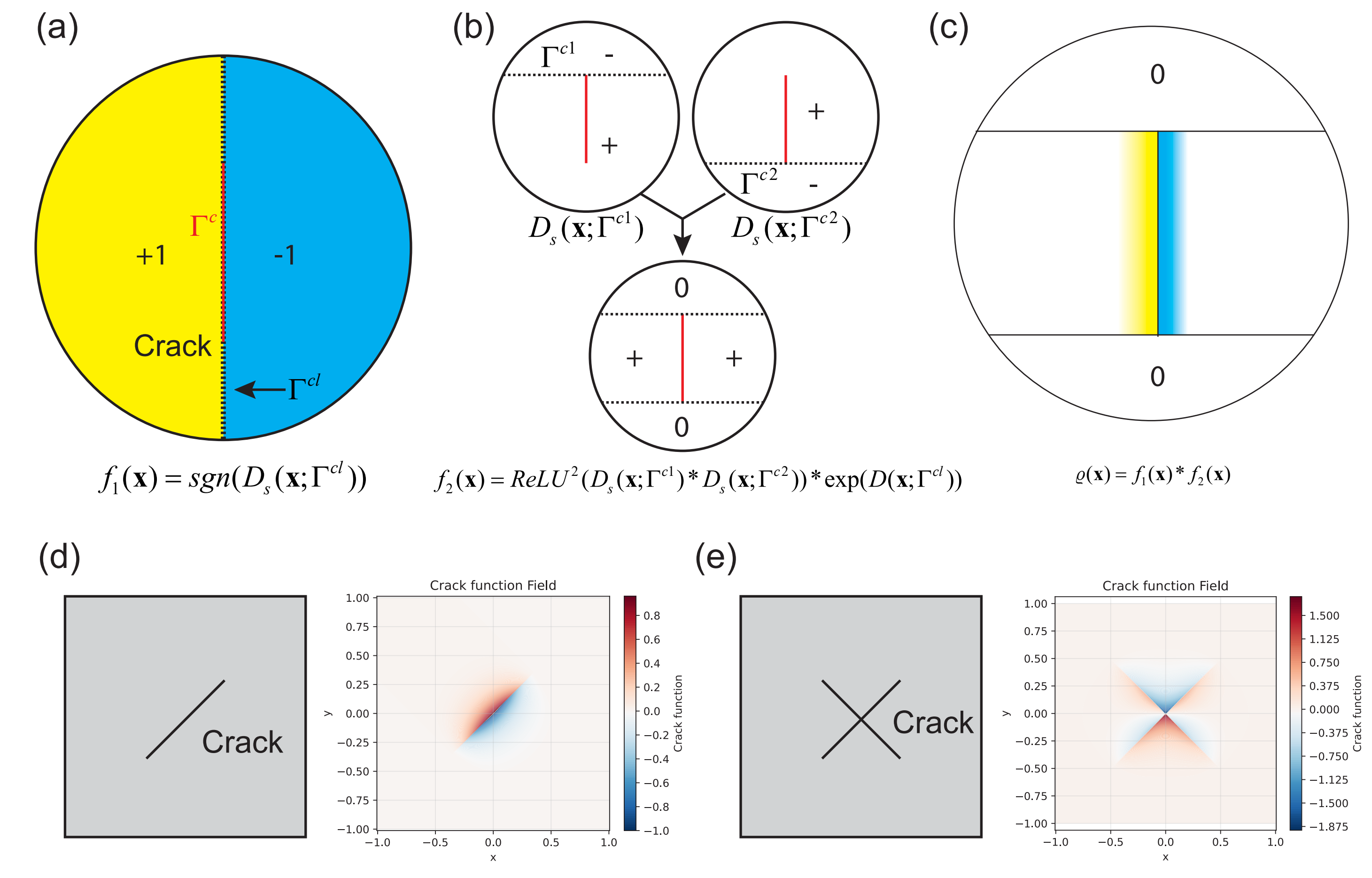

(a)
(b)
(c)
+1
-1
Crack
Γ^{c}
Γ^{cl}
Γ^{c1}
Γ^{c2}
$D_s(\mathbf{x};\Gamma^{c1})$
$D_s(\mathbf{x};\Gamma^{c2})$
$f_1(\mathbf{x}) = sgn(D_s(\mathbf{x};\Gamma^{cl}))$
$f_2(\mathbf{x}) = ReLU^2(D_s(\mathbf{x};\Gamma^{c1}) * D_s(\mathbf{x};\Gamma^{c2})) * \exp(D(\mathbf{x};\Gamma^{cl}))$
$\varrho(\mathbf{x}) = f_1(\mathbf{x}) * f_2(\mathbf{x})$
(d)
(e)
Crack
Crack function Field
Crack function
x
y
